# One-year molecular surveillance of carbapenem-susceptible *A. baumannii* on a German intensive care unit: diversity or clonality

**DOI:** 10.1186/s13756-018-0436-8

**Published:** 2018-11-26

**Authors:** Andreas F. Wendel, Monika Malecki, Robin Otchwemah, Carlos J. Tellez-Castillo, Samir G. Sakka, Frauke Mattner

**Affiliations:** 1 Institute of Hygiene, University Hospital I of Witten/Herdecke, Cologne Merheim Medical Centre, Cologne, Germany; 2 Department of Clinical Microbiology, MVZ synlab Leverkusen GmbH, Köln-Merheim, Germany; 3 Department of Anesthesiology and Operative Intensive Care Medicine, University of Witten/Herdecke, Cologne Merheim Medical Centre, Cologne, Germany

**Keywords:** Infection control, Surveillance, Bacterial typing, *Acinetobacter baumannii*

## Abstract

**Background:**

*A. baumannii* is a common nosocomial pathogen known for its high transmission potential. A high rate of carbapenem-susceptible *Acinetobacter calcoaceticus-Acinetobacter baumannii* (ACB)-complex in clinical specimens led to the implementation of a pathogen-based surveillance on a 32-bed surgical intensive care unit (SICU) in a German tertiary care centre.

**Methods:**

Between April 2017 and March 2018, ACB-complex isolates with an epidemiological link to the SICU were further assessed. Identification to the species level was carried out using a multiplex PCR targeting the *gyrB* gene, followed by RAPD, PFGE (ApaI) and whole genome sequencing (WGS, core genome MLST, SeqSphere+ software, Ridom). Additional infection prevention and control (IPC) measures were introduced as follows: epidemiological investigations, hand hygiene training, additional terminal cleaning and disinfection incl. UV-light, screening for carbapenem-susceptible *A. baumannii* and environmental sampling. Hospital-acquired infections were classified according to the CDC definitions.

**Results:**

Fourty four patients were colonized/infected with one or two (different) carbapenem-susceptible ACB-complex isolates. Fourty three out of 48 isolates were classified as hospital-acquired (detection on or after 3rd day of admission). Nearly all isolates were identified as *A. baumannii*, only four as *A. pittii*. Twelve patients developed *A. baumannii* infections. Genotyping revealed two pulsotype clusters, which were confirmed to be cgMLST clonal cluster type 1770 (*n* = 8 patients) and type 1769 (*n* = 12 patients) by WGS. All other isolates were distinct from each other. Nearly all transmission events of the two clonal clusters were confirmed by conventional epidemiology. Transmissions stopped after a period of several months. Environmental sampling revealed a relevant dissemination of *A. baumannii*, but only a few isolates corresponded to clinical strains. Introduction of the additional screening revealed a significantly earlier detection of carbapenem-susceptible *A. baumannii* during hospitalization.

**Conclusions:**

A molecular and infection surveillance of ACB-complex based on identification to the species level, classic epidemiology and genotyping revealed simultaneously occurring independent transmission events and clusters of hospital-acquired *A. baumannii*. This underlines the importance of such an extensive surveillance methodology in IPC programmes also for carbapenem-susceptible *A. baumannii*.

## Background

*Acinetobacter baumannii* is a pathogen of emerging clinical significance causing a broad range of hospital-acquired infections [1]. Its excellent capacity to survive in the hospital environment results in a high transmission propensity [2]. Additionally, rapid antibiotic resistance development makes effective therapy challenging [1]. *Acinetobacter baumannii* cannot be reliably differentiated by phenotypic or biochemical methods from other *Acinetobacter* species like *Acinetobacter pittii* (formerly genomic species 3) or *Acinetobacter nosocomialis* (formerly genomic species 13TU). These species are grouped together amongst others into the *Acinetobacter calcoaceticus-Acinetobacter baumannii* (ACB)-complex [3, 4]. However, these species differ in virulence, antibiotic resistance rates and natural habitat [1, 5]. Recent methods like MALDI-TOF/MS promise to identify the species just as accurately as molecular methods [1, 3].

Compared to other *Acinetobacter* species, *A. baumannii* is not a ubiquitous organism. It is mostly found in the hospital setting (human and environment); a natural reservoir still needs to be assessed. Healthy humans do not appear to be colonized by *A. baumannii* [1]. The population of *A. baumannii* is genetically more homogenous compared to those of *A. pittii* or *A. nosocomialis* [3, 6].

Numerous nosocomial outbreaks have been described so far, mostly with multidrug-resistant (MDR) *A. baumannii* [7]. Recent infection control and prevention (IPC) recommendations focus on multidrug-resistant *Acinetobacter* spp., taking the environment-to-patient and patient-to-patient transmission into consideration [8].

At the beginning of 2017, we retrospectively observed a high (apparently endemic) rate of carbapenem-susceptible *A. baumannii* in clinical specimens on the surgical intensive care unit in comparison to other intensive care units. Therefore, we decided to install a pathogen-based surveillance. In the present study we report on a complex increase of clonal and non-clonal *A. baumannii*, describe the local epidemiology and the IPC measures applied in a German tertiary care centre.

## Methods

### Setting

The study was conducted at a 724-bed tertiary care hospital. The surgical intensive care unit (SICU) has 32 beds (14 single rooms and 9 double rooms). Only four single patient rooms are equipped with washbasins. Adult patients from various surgical specialties are treated on the unit: general surgery, visceral surgery, vascular surgery, neurosurgery, orthopaedics, trauma, and solid organ transplantations such as kidney. The average ICU length of stay was 5.2 days in 2017. The IPC service is provided by the Institute of Hygiene. The protocol of the German healthcare-associated infection surveillance on intensive care units (ITS-KISS) was implemented on the SICU in 2012 [9].

### Microbiological sampling and analysis

A rectal and nose/throat screening for multidrug-resistant Gram-negative organisms was performed on every patient at admission and once weekly. Swabs were subsequently inoculated on chromogenic chromID® ESBL (bioMérieux) media. Additionally, tracheal secretions were sampled from intubated patients once weekly (inoculation on standard media). Identification and antimicrobial susceptibility testing (imipenem, meropenem, gentamicin, tobramycin, amikacin, ciprofloxacin, sulfamethoxazole/trimethoprim) were performed with the VITEK 2 system (VITEK 2 GN-ID and AST-N248, bioMérieux). Uncertain identification results were further investigated by MALDI-TOF (Brucker Diagnostic). Identification of *Acinetobacter* species was done to the complex level. EUCAST standards were used for interpretation. Only ACB-complex isolates being at least non-susceptible to one carbapenem and/or ciprofloxacin (in accordance with the German classification for multidrug-resistant Gram-negative organisms [10]) were reported in screening specimens at the beginning of the study in April 2017. Screening for carbapenem-susceptible ACB-complex was included in September 2017 until the end of the study because of the increasing number of isolates detected in clinical specimens. Therefore, all ACB-complex isolates growing on the cefpodoxime-containing media were reported.

### Pathogen-based epidemiology

From April 2017 onwards, microbiological data was retrieved from the laboratory surveillance information system (Hybase v.6, epiNET AG, Germany) and searched for ACB-complex isolates once daily. Only patients with an epidemiological link to the SICU were further assessed (stay on the SICU within the last month). Epidemiological data of the colonized or infected patients was collected by the infection control nurse from the patients’ clinical records and from the attending physicians or nurses.

Bacterial isolates and infections were considered as community-acquired if the collection of the specimen or the start of infection occurred on or before the 2nd day of admission. Afterwards, bacterial isolates and infections were defined as hospital-acquired. Transmission analysis was based on epidemiological data (direct room or ward contact and/or documented care by the same staff) and genetic data. Definite transmission events were defined as isolation of genetically-related isolates from two patients who were on the same ward at the same time (at least 24 h, patient-to-patient transmission) or in the same single/double room with a maximum gap of 7 days (room-to-patient transmission). Hospital-acquired infections were classified according to the Centers for Disease Control and Prevention (CDC) definitions [11]. Patients without related signs of infection were considered to be colonized.

### Molecular surveillance and genotyping

Further identification to the species level was carried out using a multiplex PCR targeting the *gyrB* gene [3]. First strain relatedness was assessed using random amplification of polymorphic DNA (RAPD) with the three primers (ERIC-1, ERIC-2 and ST272 [12, 13]) and including at least the preceding five isolates in each run. Isolates with the same banding patterns were considered to be related. Later on, pulsed-field gel electrophoresis (PFGE) was performed by using ApaI (New England Biolabs, USA). DNA separation was performed in 1% agarose in 0.5× Trisborate-EDTA (TBE) buffer in a CHEF-DR III System (Bio-Rad, LaJolla, CA, USA) using 6 V/cm for 19 h with pulse times of 5 s to 20 s. The strain relatedness was calculated with GelCompar II version 5.1 software (Applied Maths NV, Belgium) and in accordance to the criteria of Tenover et al. [14, 15].

Representative isolates of clinical and environmental samples with the same pulsotype were further assessed by whole genome sequencing (five out of eight isolates of pulsotype A and seven out of 15 isolates of pulsotype B). In short, genomic DNA was extracted with the DNeasy UltraClean Microbial Kit (Qiagen, Germany) following the manufacturer’s instructions. The library preparation using proprietary methods und subsequent sequencing using Illumina HiSeq instruments in 150 bp paired-end read mode was performed by GATC Biotech (Konstanz, Germany). Before assembly, quality control of the reads was carried out with FastQC version 0.11.5 [16]. De novo assembly was performed using Velvet (version 1.1.04) software integrated into the SeqSphere+ software version 5.0 (Ridom, Münster, Germany) using default parameters [17]. An average N50 of 582,568 bp across isolates was achieved. Afterwards, WGS-based species identification with the assembled genomes was performed using JSpeciesWS (version 3.0.18) [18]. Acquired resistance genes on assembled sequences were identified by ResFinder (version 2.1; threshold of 98% identity and minimum length of 60%) [19]. Subsequently, we applied a core genome multilocus sequence type (cgMLST) genotyping approach using the publicly available core genome MLST scheme for *A. baumannii* (2390 targets) published by Higgins et al. (SeqSphere+ software) [20]. During comparison of the allelic profile the “pairwise ignoring missing values” option was turned on. Genomes containing at least 95% of the defined cgMLST targets were included. Isolates with less than 10 different alleles in the cgMLST target gene set were considered as highly related (clonal cluster) [20].

### Infection control management and interventions

Contact (and standard) precautions were applied for every patient colonized or infected with ciprofloxacin-non-susceptible ACB-complex and/or carbapenem-non-susceptible ACB-complex (barrier nursing in two-bed room or single room, use of gowns and gloves), whereas standard precautions were applied for carbapenem- and ciprofloxacin-susceptible ACB-complex isolates. Standard or contact precautions did not change during the study period. Infection control nurses visited the SICU at least twice weekly during the whole study period.

During the surveillance of ACB-complex isolates, possible transmissions were suspected. Therefore, additional infection control measures were introduced. Hand hygiene compliance and correct use of gloves was intensified with additional standard hygiene training sessions for the health care workers (including the radiology and cleaning staff) from June to November 2017. Hand hygiene compliance observations (*n* = 200) were carried out in July and August 2017. Standard terminal cleaning and disinfection was performed once with Glucoprotamin 0.5% (Incidin plus, Ecolab Healthcare, Germany). In August and September 2017, an intensified terminal cleaning and disinfection was performed in all 23 patient’s rooms at least once as follows: disinfection two times with Glucoprotamin 0.5% and subsequently disinfection with a portable UV-light (Verilux CleanWave Sanitizing Wand, Verilux, USA) targeting complex surfaces [21]. The use of disposable patient medical equipment was encouraged. Local health authorities were informed about the clonal spread in September 2017. Environmental sampling was performed several times between June and November 2017. Sampling was conducted using moistened rayon swabs with amies transport medium (Copan, Italy). Samples were inoculated on MacConkey agar, blood agar (Oxoid, Germany) and tryptic soy broth (Merck Millipore, Germany) and incubated for a maximum of 48 h at 37 °C. Identification and susceptibility testing were performed as stated above. Sampled inanimate surfaces were as follows: patient rooms (bedside table, reusable medical equipment, washbasins, touchscreens, hand-touch sites of the endotracheal suction system), storage rooms (cupboards, shelves), electrocardiography machines, mobile x-ray systems (incl. cassettes) and disinfectant dosing systems.

### Statistical analysis

Statistical analysis was performed with PSPP 1.0.1. The Student *t* test was used for continuous normally distributed variables, the Mann-Whitney *U* test for variables that did not follow a normal distribution and the χ^2^ test or Fisher’s exact test for categorical variables.

### Nucleotide sequence accession number

Sequence reads of the strains have been deposited as a project at the European Nucleotid Archive under the accession number PRJEB27660.

## Results

### Isolate and patient characteristics

Between April 2017 and March 2018, 44 patients who had at least one epidemiological link to the SICU were found to be colonized or infected with a carbapenem-susceptible ACB-complex. Overall, 37 patients carried a fully susceptible (susceptible to all antimicrobial agents tested) ACB-complex isolate, three patients a ciprofloxacin- and sulfamethoxazole/trimethoprim-resistant ACB-complex isolate and four patients ACB-complex isolates with both phenotypes. Nearly all isolates (43 out of 48) were classified as hospital-acquired. The five community-acquired isolates (three *A. pittii*, two *A. baumannii*) were detected within 24 h; the four affected patients were not hospitalized in the preceding 30 days. The patient colonized with the two different community-acquired *A. baumannii* isolates was from a nursing home. During the same time period two patients were detected with a carbapenem-resistant ACB-complex*.*

All ACB-complex isolates, except four, were available for further identification and genotyping. Of those, two hospital-acquired and three community-acquired isolates were identified as *A. pittii*; all other isolates were identified as *A. baumannii*.

Relevant clinical and epidemiologic data of the subgroup of 36 patients with hospital-acquired colonisation/infection with carbapenem-susceptible *A. baumannii* (excluding patients with *A. pittii* or non-available ACB-complex) are displayed in Table 1. Twelve patients developed hospital-acquired infections. Seven patients died during their hospital stay. None of the patients died from an infection with ACB-complex. The anatomic site of first detection was sampled and *A. baumannii*-negative in 30 out of 36 patients during the week preceding the first detection.

**Table 1:** Epidemiologic characteristics of 36 patients with hospital-acquired *A. baumannii*

Characteristics	Value
Age (years)	
median (range)	62 (21; 80)
Gender	
female	13 (36%)
Hospital stay at first isolation (days)	
median (range)	19 (5; 62)
Source of first positive specimen^a^	
respiratory tract	14 (38.8%)
nose/throat (screening)	12 (33.3%)
rectum (screening)	7 (19.4%)
wound	4 (11,1%)
urine	2 (5.6%)
blood culture	1 (2.8%)
Infection	
pneumonia	7 (19.4%)
wound infection	2 (5.6%)
urinary tract infection	1 (2.8%)
CLABSI	2 (5.6%)
Antibiotic treatment^b^	30 (83.3%)
Surgery^b^	20 (55.5%)
Non-surgical intervention^b^	24 (66.7%)
Mechanical ventilation^b^	14 (38.8%)
Dialysis^b^	5 (13.8%)

^a^exceeds 100% as first identification was done in two different specimens in four patients; ^b^Within a maximal interval of seven days before first isolation; *CLABSI* central line associated blood stream infections

### Genotyping and transmission analysis

**Fig. 1:**
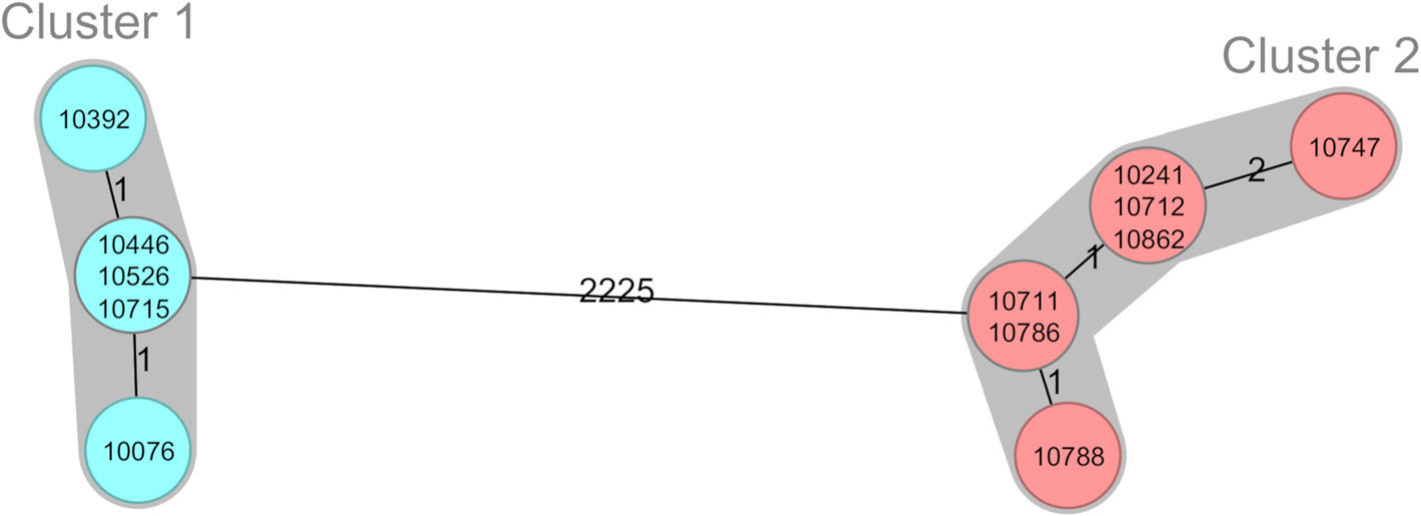
Minimum-spanning tree of the representative 12 *A. baumannii* isolates showing the genetic relationship based on the cgMLST scheme (Ridom SeqSphere+, 2359 targets). Each circle displays a single genotype and numbers on the connecting lines in between the allele difference. Clonally related genetic clusters (< 10 alleles difference) containing more than one patient are encircled in grey

Based on conventional genotyping by PFGE, we were able to show two genetically highly-related clonal clusters of fully susceptible *A. baumannii* isolates: clonal cluster 1 containing eight patients and clonal cluster 2 containing 12 patients and three environmental isolates. All other clinical and environmental isolates were distinct. Both clusters were confirmed by whole genome sequencing. The maximum distance of targets within clonal cluster 1 (cgMLST cluster type 1770, ST Oxford 753, ST Pasteur 494) were two targets, within clonal cluster 2 (cgMLST cluster type 1769, ST with Oxford or Pasteur scheme unknown) four targets and in between the clusters 2227 targets (Fig. 1).

**Fig. 2:**
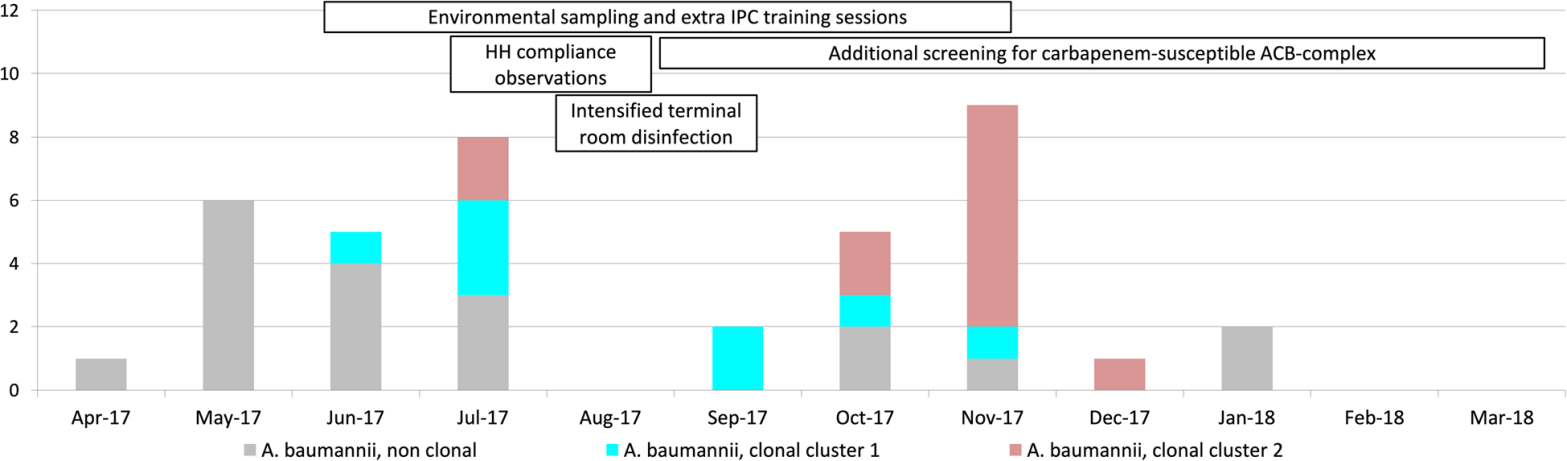
Overview of new cases with a hospital-acquired carbapenem-susceptible *A. baumannii* with an epidemiological link to the SICU (39 isolates from 36 patients). Boxes indicate additional infections control measures and their duration; HH, hand hygiene

Genetic in silico search for *bla* genes displayed the cephalosporinase-encoding *bla*_ADC-25_-like gene in both clusters, the *bla*_OXA-106_-like gene in cluster 1-isolates and the *bla*_OXA-51_ gene in cluster 2-isolates. The *bla*_OXA-106_ gene belongs to the *bla*_OXA-51_ gene subgroup [22]. Both clonal clusters emerged over a period of nearly 6 months. The epidemiologic and genetic data of the *A. baumannii*-surveillance are displayed in Fig. 2.

**Fig. 3:**
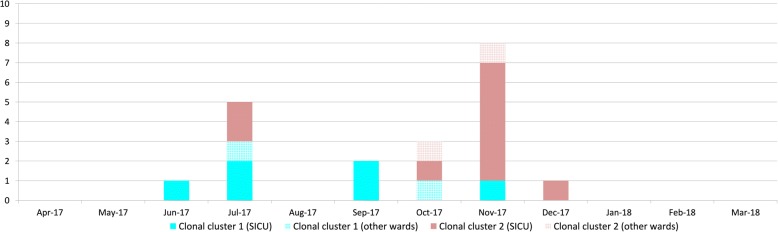
Overview of the most likely ward of acquisition of the two carbapenem-susceptible *A. baumannii* clones

Analysing spatiotemporal links based on room occupancy, we were not able to identify index patients admitted with the clones. By conventional epidemiology, nearly all transmission events were confirmed as “definite” except for one patient in each cluster where an epidemiological link to other patients or rooms could not be established. Although most definite transmissions occurred within the SICU, two patients in clonal cluster 1 and two patients in cluster 2 must have acquired the respective clone on other wards than the SICU (Fig. 3).

The two carbapenem-resistant *A. baumannii* isolates detected during the study period were neither epidemiologically nor genetically linked to each other or the other carbapenem-susceptible isolates.

### Infection control management

Observations carried out by infection control nurses revealed actions resulting in patient contamination from washbasins and vice versa. The hand hygiene compliance before patient contact, before aseptic tasks, after body fluid exposure risk, after patient contact and after contact with patient surroundings was at 75, 67, 54, 70 and 69% respectively (national reference data from the German national hand hygiene campaign AKTION Saubere Hände (ASH) from 2017: 53, 49, 74, 70 and 69% resp. [23]).

Environmental sampling revealed six ACB-complex-positive specimens out of a total of 206 specimens collected. *A. pittii*, not related to any clinical isolates, was found on one mobile x-ray system cassette. *A. baumannii* was found in a washbasin, on a fixation tape and on the pressure regulator of the endotracheal suction system at three beds. The latter isolates were genetically related to the clinical isolates of the *A. baumannii*-colonized/infected patients cared for at the time of sampling (clonal cluster 2). All mentioned positive surfaces and the pressure regulators of all endotracheal suction systems on the SICU were properly disinfected and resampled afterwards (negative). We were not able to link the acquisition of the clonal cluster strains to (non-) invasive procedures or contact to radiology.

As mentioned above, screening for susceptible *A. baumannii* was introduced in September 2017. This led to a significantly earlier detection (median: 19 days before vs. 12 days after; *p* = 0.04) of fully susceptible ACB-complex. Also, first detection of fully susceptible ACB-complex was performed significantly more from screening specimens than from clinical specimens (83.3% after vs. 22.2% before; *p* < 0.0001). All five community-acquired ACB-complex isolates were detected after the introduction of the additional screening procedures. Interestingly, no ACB-complex isolates were detected in August 2017, in February 2018 and in March 2018 (Fig. 2). However, we did not observe any change in patient numbers or screenings compliance during these months.

## Discussion

In this study, we report on a molecular and infection surveillance of carbapenem-susceptible *A. baumannii* on a surgical intensive care unit in a tertiary care centre. During the one-year study period, an initial polyclonal increase of *A. baumannii* was observed, followed by the emergence of two dominant clones.

A pathogen-based surveillance is part of the vertical approach in infection control [24]; it is mostly conducted for MDR bacteria, e.g. carbapenem-resistant *A. baumannii*, in German hospitals [9]. Hence, there is no detailed surveillance data about the occurrence and epidemiology of carbapenem-susceptible *A. baumannii*. Furthermore, we found only a few descriptions of outbreaks of carbapenem-susceptible *A. baumannii* in the literature, e.g. [25]. A recent meta-analysis reported that three quarter of *Acinetobacter* outbreaks in the literature were attributed to MDR bacteria. However, the definition of MDR is used inconsistently, and is not always in line with international recommendations [7]. The underreporting of outbreaks of non-MDR *A. baumannii* might be due to a surveillance bias, as IPC programmes put the main focus on MDR bacteria. Another reason might be a publication bias, as MDR infections are more difficult to treat and as MDR *A. baumannii* has high transmission rates [1, 7]. Moreover, MDR *A. baumannii* has become endemic in a lot of countries. The carbapenem-resistance rate exceeded 75% in invasive isolates in some south-eastern European countries in 2016 [26]. On German intensive care units, 43% of clinical isolates were resistant to imipenem in 2015 [27]. This study was conducted in a low endemic setting of carbapenem-resistant *A. baumannii* (only sporadic appearance and almost exclusively related to a hospital stay abroad).

The epidemiology of *A. baumannii* can be quite complex. Simultaneously occurring endemic and epidemic MDR *A. baumannii* clones are described in the literature, making detection and control difficult [1, 28]. The worldwide emergence of carbapenem-resistant *A. baumannii* is caused by a few successful clonal lineages [29]. The molecular epidemiology of non-MDR *A. baumannii* is less clear, but the population of non-MDR isolates is described as less homogenous [6]. Based on our results, the dynamics of transmission cannot simply be explained by an “endemic setting” of *A. baumannii.* Noteworthy, nearly all acquisitions were hospital-acquired. Moreover, the general reservoir of *A. baumannii* is unknown, as it is found almost exclusively in the hospital environment [1, 5]. Appearance and transmission of *A. baumannii* is quite common on intensive care units [7, 28] and occurs via contaminated hands after contact with the inanimate environment or colonized/infected patients. A recent review showed a positive association between infection/colonization with *A. baumannii* and exposure to rooms previously occupied by patients with *A. baumannii* [30]. In our study, we found direct and indirect evidence for both modes of transmission (patient-to-patient or environment-to-patient). The variety of environmental *A. baumannii* isolates, clonally and non-clonally related to clinical isolates, found on frequently touched surfaces, demonstrates the widespread environmental contamination and supports the importance of hand hygiene. We were not able to find definitive epidemiological links for all patients. Of course, colonized health-care workers or colonized (undetected) patients cannot be completely ruled out. It can be quite cumbersome to establish spatiotemporal links between patients, who are often transferred to several wards and units during their hospital stay. Interestingly, we also found evidence for transmissions from the SICU to and within other wards. Some electronic surveillance systems can help to detect patient contacts, e.g. the Hybase software used in this study. However, they fail if several patients from different wards are affected or in cases of indirect transmissions (room contact). Easy to use software solutions are lacking to analyse and visualize complex patient transfers.

The transmissions of the clonal strains were stopped with a combination of standard hygiene measures, intensified environmental cleaning and disinfection and rectal screening for carbapenem-susceptible ACB-complex. Contact precautions were only applied to a minority of patients as stated above. The expanded screening was introduced in the middle of the study period and resulted in a significantly earlier detection of ACB-complex. Before the introduction, community-acquired isolates might have been misclassified as hospital-acquired due to the late detection. Neither the sensitivity of patient screening for (MDR) *A. baumannii* colonization nor the ideal screening loci are well known. Recent studies showed a low sensitivity of detection in colonized patients [31] or demonstrated that perirectal screening might be more appropriate than rectal screening for *A. baumannii* [32]. Nevertheless, in our opinion the combination of three screening loci (rectal and nose/throat) and subsequent inoculation on a cefpodoxime-containing media, as performed in our study, reach a sufficient sensitivity.

We also applied UV light to disinfect complex surfaces [21] in combination with two times standard cleaning and disinfection over a period of 2 months. As rooms were blocked for hours during this procedure, these additional measures were stopped. Therefore, the impact of additional environmental cleaning and disinfection remains unclear in our study. However, intensified room cleaning and disinfection was described as an effective IPC measure during outbreak periods [33]. We also observed a relevant environmental contamination, especially on difficult-to-clean regulators of the endotracheal suction system. As nearly all of them were contaminated with skin flora and other pathogens (e.g. *Klebsiella pneumoniae, Enterococcus faecium*), they might have been a reservoir of transmission. As there is no dedicated cleaning staff for terminal cleaning and disinfection of medical products in our hospital during the late and night shifts, this task is assigned to the nurse at times of patient-to-nurse ratios of sometimes 3:1 or worse. This ratio is not unusual on German intensive care units [34].

The *A. baumannii* infection rate of approx. 50% was described during outbreaks, with no difference between MDR- and non-MDR infected patients [7]. In our cohort, we observed a hospital-acquired *A. baumannii* infection rate of 33.3% (12 out of 36 patients). The majority of our patients showed the relevant risk factors for colonization/infection with *A. baumannii,* already well described for MDR *Acinetobacter,* such as prolonged hospital stays at an ICU, mechanical ventilation or prior antimicrobial therapy [7, 28]. Selective pressure of broad-spectrum penicillins or third-generation cephalosporins, both favoured in our hospital, might have played a decisive role.

We encountered some pitfalls of such surveillance. A proper and reliable (molecular) identification of the isolates to the species level is needed, as the different ACB-complex species differ in their hospital epidemiology. Especially *A. baumannii* and to a certain extent *A. nosocomialis* are known to cause outbreaks in hospital settings [1, 5]. In our opinion, it is important to consider microbiological results after the patients’ transfer to other wards. We would have missed seven patients if we had only included specimens collected on the SICU. Hence, a hospital-wide surveillance appears more appropriate to identify possible transmission pathways, but requires extended IPC resources. Furthermore, a typing method with high discriminatory power is crucial. In our opinion, RAPD and PFGE are suitable methods for typing of *A. baumannii*. However, in times of next generation sequencing (NGS), a “random access” database may be more appropriate. For example, a gene-by-gene approach with an accepted allele scheme (cgMLST) can be used over longer periods [35]. The application of a cgMLST scheme was recently demonstrated by Willems et al. in an outbreak of *A. baumannii* [36]*.*

There are a few limitations to this study. First, we only analysed one isolate per resistance pattern per patient. Patients colonized/infected with more than one strain might have remained undetected. Secondly, a formal case–control study to determine risk factors for carriage of carbapenem-susceptible *A. baumannii* was not performed. However, our aim was to describe the surveillance results and the molecular epidemiology. Thirdly, we only applied NGS for isolates that were highly related using PFGE and RAPD for economic reasons. Related isolates showing different PFGE/RAPD patterns, though unlikely, might have gone unnoticed.

## Conclusions

A molecular and infection surveillance of ACB-complex based on identification to the species level, classic epidemiology and genotyping revealed simultaneously occurring independent transmission events and clusters of hospital-acquired *A. baumannii*. This underlines the importance of such an extensive surveillance methodology in IPC programmes also for carbapenem-susceptible *A. baumannii*.

## Abbreviations

ACB: *Acinetobacter calcoaceticus-Acinetobacter baumannii*
cgMLST: Core genome multilocus sequence type
CLABSI: Central line associated blood stream infections
HH: Hand hygiene
IPC: Infection prevention and control
ITS-KISS: Intensivstation-Krankenhaus-Infektions-Surveillance-System = German national nosocomial infections surveillance on intensive care units
MDR: Multidrug-resistant
NGS: Next generation sequencing
PFGE: Pulsed-field gel electrophoresis
RAPD: Random amplification of polymorphic DNA
SICU: Surgical ICU
WGS: Whole genome sequencing
